# Additive and Transcript-Specific Effects of KPAP1 and TbRND Activities on 3′ Non-Encoded Tail Characteristics and mRNA Stability in *Trypanosoma brucei*

**DOI:** 10.1371/journal.pone.0037639

**Published:** 2012-05-21

**Authors:** Sara L. Zimmer, Sarah M. McEvoy, Sarita Menon, Laurie K. Read

**Affiliations:** Department of Microbiology and Immunology, School of Medicine and Biomedical Sciences, University at Buffalo, State University of New York, Buffalo, New York, United States of America; Texas A&M University, United States of America

## Abstract

Short, non-encoded oligo(A), oligo(U), or A/U tails can impact mRNA stability in kinetoplastid mitochondria. However, a comprehensive picture of the relative effects of these modifications in RNA stability is lacking. Furthermore, while the U-preferring exoribonuclease TbRND acts on U-tailed gRNAs, its role in decay of uridylated mRNAs has only been cursorily investigated. Here, we analyzed the roles of mRNA 3′ tail composition and TbRND in RNA decay using cells harbouring single or double knockdown of TbRND and the KPAP1 poly(A) polymerase. Analysis of mRNA abundance and tail composition reveals dramatic and transcript-specific effects of adenylation and uridylation on mitochondrial RNAs. Oligo(A) and A-rich tails can stabilize a proportion of edited and never-edited RNAs. However, non-tailed RNAs are not inherently unstable, implicating additional stability determinants and/or spatial segregation of sub-populations of a given RNA in regulation of RNA decay. Oligo(U) tails, which have been shown to contribute to decay of some never-edited RNAs, are not universally destabilizing. We also show that RNAs display very different susceptibility to uridylation in the absence of KPAP1, a factor that may contribute to regulation of decay. Finally, 3′ tail composition apparently impacts the ability of an RNA to be edited.

## Introduction

The kinetoplastid parasites include several human pathogens such as *Leishmania* spp., *Trypanosoma cruzi* and *T. brucei*, which cause leishmaniasis, Chagas' disease, and African sleeping sickness, respectively. Kinetoplastid parasites are transmitted between mammalian hosts by insect vectors, and as such face very dramatic environmental changes during their life cycles. Thus, elaborate schemes of gene regulation, primarily effected at post-transcriptional levels, are invoked for both proliferative growth in insect and mammalian hosts, and to instigate the developmental changes required to complete the life cycles of these parasites. One dramatic example of regulation during the life cycle is in the mitochondria of *T. brucei*, which undergoes extensive changes in gene expression, physiology, and morphology as the organism cycles between its insect and mammalian hosts [1], [2], [3], [4].

A hallmark of kinetoplastid mitochondria is the process of uridine (U) insertion/deletion RNA editing. RNA editing, directed by small guide RNAs (gRNAs) and catalyzed by a multiprotein complex called the editosome or RNA editing core complex (RECC), is required to generate the mature, translatable form of many mRNAs [5], [6], [7], [8]. In *T. brucei*, 12 of the 18 mitochondrially-encoded mRNAs undergo some degree of editing (the other six mRNAs are termed “never-edited”). Differences in the degrees of editing of specific RNAs in different life cycle stages of *T. brucei* suggest that regulation of the editing process may contribute to changes in the abundance of mature mitochondrial RNAs [1], [9], [10], [11], [12].

In addition to editing, regulation of RNA levels in trypanosome mitochondria also appears to occur via RNA turnover. For example, the abundance of mature monocistronic mRNAs, including those that do not undergo editing, often varies dramatically between human bloodstream form (BF) and insect procyclic form (PF) life cycle stages [12], [13], [14], [15], [16], suggesting that the stability of specific RNAs is regulated developmentally and/or in response to external or internal signals. RNA stability appears to be linked to non-encoded nucleotide extensions, or tails, on the 3′ ends of mRNAs. Tails on trypanosome mitochondrial mRNAs are classified into two categories. One class is comprised of extensions that are 40 to several hundred nucleotides long that are suggested to function in the interface between editing and translation, but have not been shown to be regulators of mRNA stability [17]. The other class of extensions is shorter, as short as one nucleotide, and are more ubiquitous, decorating pre-edited, partially edited, edited and never-edited mRNAs. These “short” tails are often described as “poly(A) tails”, but evidence suggests that short tails are often comprised of both adenosine (A) and U [17], [18], [19], [20]. Moreover, limited sequencing suggests that both the length and A/U ratio of short 3′ tails may sometimes differ between different RNAs, showing some transcript specificity [17], [19], [20]. In contrast to the long tails, these heterogeneous short tails apparently function in the regulation of transcript stability. For example, KPAP1 is the primary mitochondrial poly(A) polymerase responsible for adding A to both the short and long tails on mRNA 3′ ends [21]. When KPAP1 is depleted and A addition to 3′ transcript ends is curtailed, many edited and never-edited mRNAs are observed in lower abundance, while some pre-edited transcripts appear to build up, suggesting that the presence of a poly(A) or A-rich tail may differentially affect the stabilities of these transcripts [21]. Furthermore, in *in vitro* degradation assays in partially purified mitochondrial extracts, untailed and oligo(A) tailed RNA substrates are differentially susceptible to decay, in a way that is consistent with results of the *in vivo* analysis in KPAP1 depleted cells [22], [23]. mRNAs are also decorated by RET1, a terminal uridyltransferase that adds Us to mRNAs, in addition to adding oligo(U) tails to mitochondrial rRNAs and gRNAs. When RET1 is depleted, the never-edited mRNAs MURF1 and ND1 are present at much higher levels, suggesting that U addition serves to destabilize these RNAs [17]. While depletion of RET1 is a good way to investigate the effects of uridylation on RNA abundance for never-edited transcripts, the fact that its depletion interferes with the process of editing due to its effects on gRNAs prevents us from studying the possible effects of RET1 depletion on the stability of transcripts that undergo editing. The KPAP1 and RET1 depletion studies described above have shown that tail removal results in changes in mRNA abundances. However, we still lack a comprehensive picture regarding the general or transcript-specific impact of adenylation versus uridylation, or whether the organism is able to alter these two activities to effect changes in relative mRNA levels.

Several exoribonucleases have been described in *T. brucei* mitochondria, but functional studies are not consistent with a role for these enzymes in general mRNA decay. Two of the four mitochondrial proteins with known exoribonuclease domains, KREX1 and KREX2, are U-specific components of the editosome whose activities are apparently devoted to U deletion RNA editing [8]. A third mitochondrial exoribonuclease, TbDSS-1, functions in RNA surveillance and has pleitotropic effects on mitochondrial RNA stability, but apparently does not appear to be involved in bulk mRNA turnover [24], [25]. Finally, TbRND is a novel RNase D family 3′ to 5′ exoribonuclease whose activity is confined to U polymers [26]. Depletion and overexpression studies demonstrated that TbRND plays a role in gRNA metabolism. While our previous study did not reveal a role for TbRND in mRNA metabolism, such an effect could have been missed due to inadequate depletion of the enzyme by RNAi or secondary effects of other enzymes.

In this study, we sought to better clarify the roles of uridylation and adenylation in mRNA stability and determine whether TbRND could impact mRNA stability under conditions where RNAs are preferentially uridylated. We analyzed RNA levels and 3′ tail composition in cells depleted of KPAP1, TbRND, or both enzymes simultaneously. Our results indicate that mitochondrial RNAs in wild type cells often harbor a mixed population of 3′ tails, and the depletion of KPAP1 leaves transcripts differentially susceptible to uridylation. Furthermore, although the appearance of oligo(U) tails on mRNAs was common upon KPAP1 depletion, these uridylated transcripts do not appear to be direct targets of TbRND. TbRND depletion in the KPAP1 RNAi background did result in both positive and negative indirect effects on transcript abundance and 3′ tail characteristics that were transcript specific. Finally, our results suggest that 3′ tail composition can impact the ability of an RNA to enter the RNA editing pathway. Overall, these studies reveal that both the composition of mitochondrial mRNA 3′ tails and the impacts of these *cis*-acting sequences on mRNA stability are more complicated that previously appreciated.

## Materials and Methods

### cDNA cloning and plasmid construction

For expression of tetracycline (tet)-inducible RNAi, nucleotides 1 to 687 nt of the KPAP1 gene (Tb11.02.5820) amplified using primers 5′-GCGGATCCATGAGAAAGTTTTCAGCTTTTCG-3′ and 5′-GCGGAT CCTGGAAGACGCAAAGGGATGTC-3′, were cloned into the p2T7-177 plasmid [27] at the BamHI restriction site internal to opposing T7 promoters to generate p2T7-177KPAP1. This same fragment of KPAP1 was also cloned into the BamHI site of p2T7-177TbRND [26] to generate p2T7-177KPAP1TbRND.

### *T. brucei* cell culture, transfection and induction, and mitochondrial extract preparation

PF *T. brucei* strain 29-13 (from Dr. George A.M. Cross, Rockefeller University), which contains integrated genes for the T7 RNA polymerase and the tet repressor, were grown in SDM-79 media supplemented with 10% fetal bovine serum (FBS) as indicated previously [28]. To generate a tet-inducible clonal KPAP1 and KPAP1/TbRND RNAi cell line, NotI-linearized p2T7-177KPAP1 or p2T7-177KPAP1TbRND was transfected into 29-13 cells, resulting in phleomycin-resistant polyclonal cultures. Clones were obtained by limiting dilution, and induced with tet at 1×10^6^ cells/ml, with cells harvested at day 3 for RNA collection. RNA was collected in the same way for the TbRND RNAi cell line [26]. In all cases, cells were induced at 2.5 µg/ml tet, and for growth curves cells were induced at a concentration of 1×10^6^ cells/ml and diluted as necessary every 24–48 hours. Values from three or four independent growth experiments were averaged to generate growth curves. To isolate mitochondria for subsequent RNA extraction, cells from 1 L of culture grown from each RNAi cell line, both with and without tet induction of RNAi, were harvested and the isolation was performed as described [29].

### RNA extraction

To collect RNA from mitochondria from uninduced and induced TbRND RNAi and KPAP1 RNAi, and KPAP1/TbRND RNAi cells, the acid guanidinium thiocyanate-phenol-chloroform method of extraction was used on the isolated mitochondria [30], followed with an additional organic extraction. Mitochondrial RNA samples were run on 6% polyacrylamide and visualized to confirm depletion of non-mitochondrial ribosomal RNA and consistency between samples. A separate biological replicate mitochondrial purification and RNA extraction was also performed, and a limited amount of edited MURF2 circular RT-PCR was performed on these samples to ensure the validity of observed differences in the KPAP1 and KPAP1/TbRND RNAi lines.

### qRT-PCR

For qRT-PCR, four µg of RNA was treated with a DNase kit (Ambion) to remove any residual DNA. RNA was reverse transcribed and amplified using a MyiQ single-color real-time PCR detection system as described [31] using primers specific to pre-edited, edited, and pre-processed RNAs described in [21], [31], [32], [33]. RNA levels represent the mean and standard deviation of 3 or more determinations.

### Circular RT-PCR

Mitochondrial RNA was DNase treated with a DNase kit (Ambion), followed by extraction with phenol/chloroform/isoamyl alcohol (25∶24∶1) and precipitation. Ten µg of the DNase treated RNA was circularized in a 400 µl reaction volume with 80 units of T4 RNA ligase (Epicentre) with the included buffer, a final concentration of 20 µM ATP, and 80 units of RNase inhibitor at 4°C overnight. After another phenol/chloroform/isoamyl alcohol (25∶24∶1) extraction and precipitation, the RNA was resuspended in H_2_O and 1 µg was reverse transcribed using SuperScript III (Invitrogen) and one of the following gene-specific oligonucleotides: 5′-CCCATAAAAAATACAAATCATAGACTG-3′ for ND4; 5′-TTATTCAAAAGAAGCTCTCCGTCG-3′ for pre-edited RPS12; 5′-CAAAACGTAAACAACAACCATA-3′ for edited RPS12; and 5′-ATCAAACCATCACAATATAAAATCATATG-3′ for edited MURF2, according to the manufacturer's instructions. For PCR, weighted dNTPs were used (the 10X dNTP mix consisting of 40 mM total dNTPs, with the concentration of dGTP and dCTP being half that of dTTP and dATP). For amplification of ND4 RNA 3′ tails, primers 5′-GTATTTATGTCAATATCAATATCAACTATAG-3′ and 5′-GTAACAATTAACAATATAAAATTTATAC-3′ at 0.2 µM final concentration were used in a 100 µl final volume with 20% of the gene-specific RT reaction and Taq polymerase in a 40 cycle reaction. For amplification of pre-edited RPS12 RNA 3′ tails, primers 5′-GAAACATCGTTTAGAAGAGATTTTAGA-3′ and 5′-CCACTCAAAAAATCCTCGCC-3′ at 0.2 µM final concentration were used in a 100 µl final volume with 12.5% of the gene-specific RT reaction and Taq polymerase in a 40 cycle reaction. Partially-edited RPS12 was amplified with the same antisense primer as pre-edited RPS12, but using the sense primer 5′-ATTATACACGTATTGTAAGTTAGATTTAGA-3′ and 12.5 percent of both RPS12 RT reactions. Finally, for amplification of edited MURF2 RNA 3′ tails, primers 5′-TCAGTTTTGTTTAACACAGTTATTATC-3′ and 5′-CAAAGCACAAAAATAAAACTAAATTAAAA-3′ at 0.1 µM final concentration were used in a 100 µl final volume with 12.5% of the gene-specific RT reaction and Taq polymerase in a 40 cycle reaction. Products of the entire PCR reaction were precipitated with ethanol and used in a 40 cycle nested PCR reaction with 0.1 µM fresh addition of the same sense primer and 0.2 µM concentration of the antisense primer 5′-TAAAACTAAATTAAAACAACCAAAC-3′. In all cases, PCR products were purified with the Illustra GFX PCR purification kit (GE Healthcare) or phenol/chloroform/isoamyl alcohol (25∶24∶1) extraction, precipitated, and resuspended in 6 µl H_2_O. A third of this yield was ligated into TOPO pCR2.1, and used to transform Top10 *E. coli* cells.

### mRNA 3′ tail analysis

15 clones for each uninduced RNAi cell line, 15 clones from the induced TbRND RNAi cells, and 30 clones from the induced KPAP1 and KPAP1/TbRND RNAi cell lines were selected for sequencing. Sequences from all uninduced cultures were combined to generate the sequences of the “Control” population. Random C or G nucleotides interspersed in the sequences (consisting of less than 1% of the total nucleotides) were eliminated from the subsequent analysis, as they occurred too seldom to determine whether they were indeed part of the tail sequence rather than a PCR or sequencing artifact. General characteristics of tails were calculated in an Excel spreadsheet, and dot plots were generated with a dot plot generator created by Tatsuki Koyama at Vanderbilt University. Tails were considered homopolymeric if from the second nucleotide to the end of the tail, they consisted of only one nucleotide. A paired, one-tailed Student's t-test was used to verify the shortening of U tails on pre-edited RPS12.

## Results

### Effects of KPAP1/TbRND co-depletion on growth and mitochondrial RNA levels

We previously reported that depletion of the TbRND exoribonuclease to approximately 40% of normal levels lead to increased gRNA oligo(U) tail length, but did not have significant effects on levels of mitochondrial mRNAs as determined by qRT-PCR [26]. However, in these studies, we could not definitively rule out a direct effect of TbRND on mRNA decay because this relatively modest level of depletion may not have been adequate to generate substantial changes in mRNA abundance. In addition, the effects of reduced TbRND activity on mRNAs may have been masked by the activities of other exoribonucleases, such as those that target oligo(A) or A-rich tails. Because TbRND is a 3′ to 5′ exoribonuclease with a preference for oligo(U), its potential to effect mRNA decay would presumably be sensitive to mRNA 3′ tail composition. Reported trypanosome mitochondrial mRNA tails range from solely A, to A/U, to solely U [12], [17], [18], [19], [20], [21]. Thus, we sought to modulate mRNA 3′ tail composition *in vivo*, and test whether alterations in the percent of U residues in 3′ tails impact the ability of TbRND to degrade mRNAs. We reasoned that depleting the major mitochondrial poly(A) polymerase (KPAP1), while leaving the mRNA TUTase, RET1, intact would lead to increased U content in mRNA 3′ tails. This change in average mRNA tail U content might permit us to detect an effect of TbRND on mRNA decay patterns. Additionally, these experiments have the potential to provide insight into the interplay between mRNA 3′ adenylation and uridylation and to reveal whether different mRNAs are differentially sensitive to these modifications.

**Figure 1:**
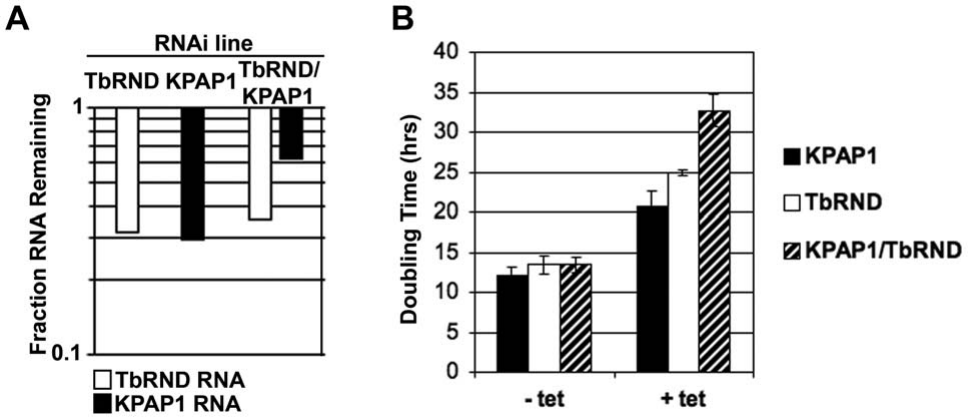
Growth of single and dual KPAP1 and TbRND RNAi cell lines. A. Extent of target RNA depletion. Relative transcript levels in tetracycline (tet) - induced RNAi cells compared to levels prior to RNAi induction, as determined by quantitative RT-PCR normalized to 18S RNA. B. Doubling time before and after tet induction of RNAi for TbRND, KPAP1, and dual-TbRND/KPAP1 RNAi cell lines. Doubling time was calculated using cell numbers on days 3 to 10 following tet induction, the time period when growth was linear for all strains in all conditions when plotted on a logarithmic scale.

To answer these questions, we generated procyclic form cells lines harboring inducible dual KPAP1/TbRND RNAi, as well as single TbRND and KPAP1 RNAi lines for comparison. Upon tet induction, TbRND mRNA was depleted to 30–35% of normal levels in both the single and double RNAi lines (Fig. 1A). KPAP1 mRNA was reduced to 30% of normal levels in the single RNAi line and 60% of normal in the double knockdown line (Fig. 1A). All three knockdown lines displayed growth defects, consistent with previously reported effects of KPAP1 and TbRND depletion [21], [26]. We determined the cell doubling times for both single and double RNAi lines in the period from 3 to 10 days post-induction. Cell doubling time increased by a factor of about two under conditions of KPAP1 or TbRND depletion, and by a factor of approximately 2.7 in the KPAP1/TbRND co-RNAi cells (Fig. 1B). Thus, growth is more severely curtailed in the co-depleted strain, even though the remaining KPAP1 levels remain higher in the co-depleted line than in the single KPAP1 knockdown.

**Figure 2:**
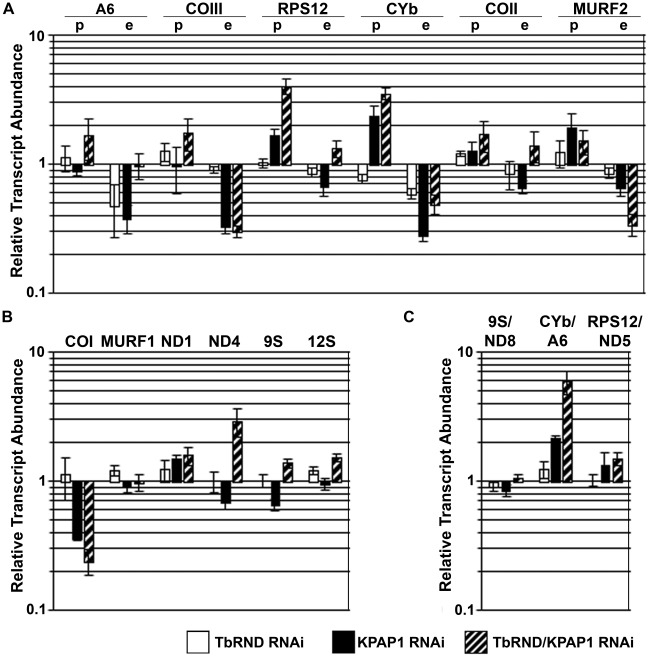
Mitochondrial transcript abundances in TbRND, KPAP1, and dual-TbRND/KPAP1 RNAi cell lines. Relative transcript levels of indicated transcripts compared to levels prior to RNAi induction, as determined by quantitative RT-PCR normalized to 18S RNA. A. Edited transcripts. p, amplification with primers specific to the pre-edited form of the transcript; e, amplification with primers specific to the edited form. B. Never-edited transcripts. The abundance values for 9S and 12S rRNAs for the TbRND RNAi were published previously [26], but included here for the purposed of comparison. C. Pre-processed transcripts. Amplification with primers spanning two adjacent genes.

We next asked whether altered mRNA levels could be a factor contributing to the slow growth of the KPAP1/TbRND RNAi cell line by comparing the levels of mitochondrial RNAs in cells depleted of KPAP1 or TbRND to those simultaneously depleted of both enzymes (compare hatched bars to black or white bars in Fig. 2). Since it is unlikely that depletion of either KPAP1 or TbRND would affect relative transcriptional rates, we putatively linked changes in abundance with changes in stability for the purposes of this study. Equating RNA abundance with stability is conventional in this field; justified by the lack of evidence for transcriptional control, presence of polycistonic RNAs [14], [15], [34], [35], and inability to effectively inhibit the mitochondrial RNA polymerases. We analyzed several classes of mitochondrial mRNAs, including pre-edited, edited, and never-edited, as well as ribosomal and dicistronic RNAs, by quantitative RT-PCR (qRT-PCR). For these analyses, we used mRNA collected before and 3 days after tet induction, a time point prior to an evident growth defect. The relative levels of qRT-PCR products from the KPAP1 RNAi cell line for each transcript in induced vs. uninduced cells are shown in Fig. 2. For comparison purposes, we also show results for TbRND RNAi that were previously reported but not shown in [26]. In cells depleted of TbRND, we observed almost no changes in mRNA levels, with the exception of edited A6 and CYb RNAs, which were decreased to about 50% and 55% of normal levels, respectively (white bars, Fig. 2). If TbRND were directly degrading these RNAs we would have expected an increase, rather than a decrease in their levels. Thus, TbRND apparently affects the levels of some mitochondrial RNAs indirectly. In contrast to TbRND depletion, KPAP1 depletion markedly affected mitochondrial RNA levels (black bars, Fig. 2). Our results mirror closely those previously published for a different KPAP1 RNAi cell line in which KPAP1 RNA was depleted to approximately the same amount [21]. One exception is that we did not observe changes in the abundance of the never-edited transcripts, MURF1 and ND1, while depletion in the 0.5 fold range was observed previously (Fig. 2B). In addition, both studies demonstrate a 1.5–2 fold increase in abundance of pre-edited CYb and MURF2 RNAs upon KPAP1 depletion; however, we detected a similar effect on pre-edited RPS12 RNA that was not observed by Etheridge, *et al.* [21] (Fig. 2A). Most importantly, in both this and the previous study [21], edited forms of all transcripts tested were reduced to between 20 and 70% of wild type levels, suggesting that loss of KPAP1-catalyzed adenine addition destabilizes these transcripts (Fig. 2A). We also observed a slight decrease in mitochondrial rRNAs upon KPAP1 depletion, consistent with previous results (Fig. 2C). In addition, we analyzed the abundance of three dicistronic precursor transcripts in KPAP1 depleted cells. Two of these were unaffected, while transcripts spanning CYb and A6 RNAs were increased two-fold upon KPAP1 depletion (Fig. 2C).

Having analyzed mitochondrial RNA levels in TbRND and KPAP1 single knockdowns, we next analyzed the abundance of the same RNAs in the dual knockdown line. We observed numerous changes in mRNA levels in the KPAP1/TbRND line compared to the single knockdown lines, and these changes were transcript-specific in both magnitude and direction. Because most edited RNAs and some never-edited RNAs are destabilized by KPAP1 depletion, we first asked whether TbRND is the enzyme that degrades edited RNAs lacking an oligo(A) tail. If this were the case, we would expect edited RNAs to increase in abundance in the dual knockdown compared to the KPAP1 RNAi line. We do observe this trend for edited A6, RPS12, COII, and to a lesser extent CYb (Fig. 2A; compare hatched to black bars). However, we see no increase in edited COIII RNA and a two-fold decrease in edited MURF2 RNA in the dual knockdown compared to the single knockdowns (Fig. 2A). Likewise, we observe no restoration of never-edited COI RNA levels by depletion of TbRND in the KPAP1 RNAi background (Fig. 2B). Thus, we conclude that TbRND is unlikely to be the enzyme universally responsible for degrading edited and never-edited RNAs upon KPAP1 depletion. With regard to the other mitochondrial RNA populations, the impact of KPAP1/TbRND co-depletion is relatively modest, with a few striking exceptions (Figs. 2A–D). Pre-edited RPS12 and never-edited ND4 RNAs are increased two- and three-fold, respectively, above the levels in the KPAP1 single knockdown (Figs. 2A and B). Similarly, the dicistronic precursor RNA spanning CYb-A6 is increased three-fold in the dual knockdown line compared to the KPAP1 single RNAi line (Fig. 2D). From these data, we conclude that TbRND has pleitropic and transcript-specific effects on mitochondrial mRNA levels. These effects are sensitive to changes in KPAP1 levels, and thus may be responsive to mRNA 3′ tail composition.

### Effects of KPAP1, TbRND, and KPAP1/TbRND depletion on ND4 RNA 3′ tail composition

**Figure 3:**
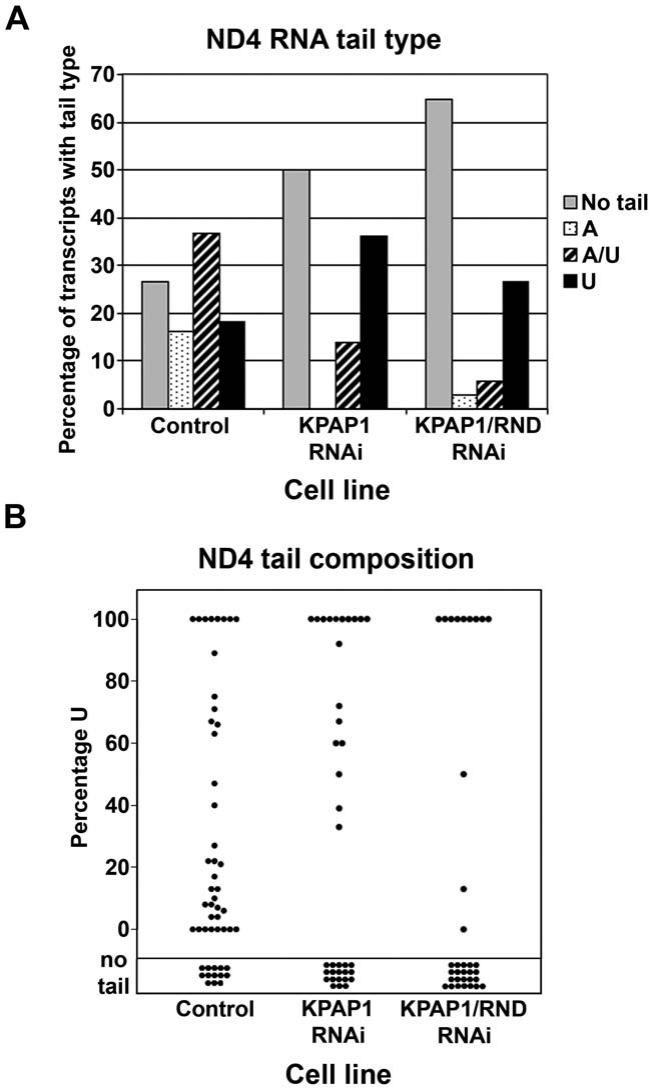
Analysis of ND4 RNA non-encoded tails. A. Percentage of the total population that possesses each tail type in cells collected 3 days post-induction of RNAi and in uninduced cells. Total number of tails in each of the three populations ranged from 34 to 49. B. For each non-encoded tail obtained from each cell type, the percentage U in the tail was calculated and plotted. “Control population” is comprised of sequences derived from RNA of *uninduced* cultures of the TbRND, KPAP1, and KPAP1/TbRND RNAi cell lines.

In an effort to identify characteristics of 3′ tails on mRNAs whose abundance is altered by TbRND depletion in the KPAP1 background, we directly investigated 3′ tail compositions in the differing cell lines. We began by examining the never-edited ND4 RNA, which increased in abundance upon dual KPAP1/TbRND depletion. Specifically, ND4 RNA levels were unchanged upon TbRND depletion, decreased 30% upon KPAP1 depletion, and increased 3-fold over uninduced cells when TbRND was depleted in the KPAP1 RNAi background (Fig. 2B). The 3′ ends of ND4 RNAs were sequenced using circular RT-PCR (cRT-PCR), which involves circularizing the total population of RNAs with RNA ligase, reverse transcribing with a gene-specific primer, and generating PCR products using gene-specific sets of nested PCR primers positioned to amplify across the ligated region of the molecule containing the 5′ and 3′ ends. The resulting cDNAs are then cloned and sequenced. To obtain the largest, most heterogeneous population of ND4 cRNAs from the circularized molecules, we started with mitochondrial RNA. Mitochondria were collected from all cell lines concurrently and all RNA populations were treated equivalently; thus, RNA populations from each cell line can be directly compared. Because numerous reports indicate that mitochondrial RNAs can differ with respect to their 3′ tail sequences, we began by analyzing ND4 3′ tail composition in the control RNA population, consisting of the combined RNAs from all uninduced cell lines, after verifying that these populations did not significantly differ from each other (Dataset S1). Surprisingly, we found that >25% of ND4 RNAs in the steady state population lack a 3′ tail altogether (Fig. 3). Tails consisting of solely A or solely U respectively constitute 16 and 18% of the population, and the remaining 37% of ND4 tails an A/U mixture. Of the 75% of RNAs with a 3′ tail, the percentage of U in the tails ranged from 4–89%, with a slight bias towards more A-rich tails (Fig. 3B, Fig. S1).

Upon depletion of TbRND alone, we observed no obvious changes in 3′ tail composition or length in any RNA examined (Dataset S1). When KPAP1 is depleted, we observe on ND4 RNAs an elimination of A tails, a decrease in A-rich tails, and an increase in transcripts with U tails or lacking tails, as a proportion as the total, as might be expected during depletion of an adenylating enzyme (Fig. 3A). This proportional change in tail composition is correlated with a slight decrease in ND4 RNA stability (Fig. 2B). Together, these data suggest that the destabilized ND4 RNAs were a subpopulation of ND4 RNA that normally is stabilized by an A or A-rich tail. Because U-tailed RNAs are a substrate for TbRND [26], the increased proportion of ND4 RNAs with U tails and U-rich tails in KPAP1 RNAi cells may be susceptible to attack by TbRND, thereby accounting for the increase in ND4 RNA abundance upon TbRND depletion in the KPAP1 depleted background (Fig. 2B). To test this, we examined ND4 tails in the dual knockdown line. If the above scenario is correct, we expect that upon TbRND/KPAP1 co-depletion, U-tailed and possibly U-rich RNAs would accumulate and constitute a greater percentage of the ND4 RNA population. In fact, it appears that the opposite is true. U-tailed RNAs decrease from 36% to 25% of the total population, while untailed RNAs increase from 50% to 65% of the population in the dual knockdown line compared to KPAP1 knockdowns. Therefore, oligo(U) tails on ND4 RNAs, which increase in abundance following KPAP1 depletion, do not appear to be TbRND substrates. These results suggest that TbRND depletion causes accumulation of ND4 mRNA by an indirect mechanism.

### Effects of KPAP1, TbRND, and kPAP1/TbRND depletion on pre-edited and partially edited RPS12 3′ tail composition

**Figure 4:**
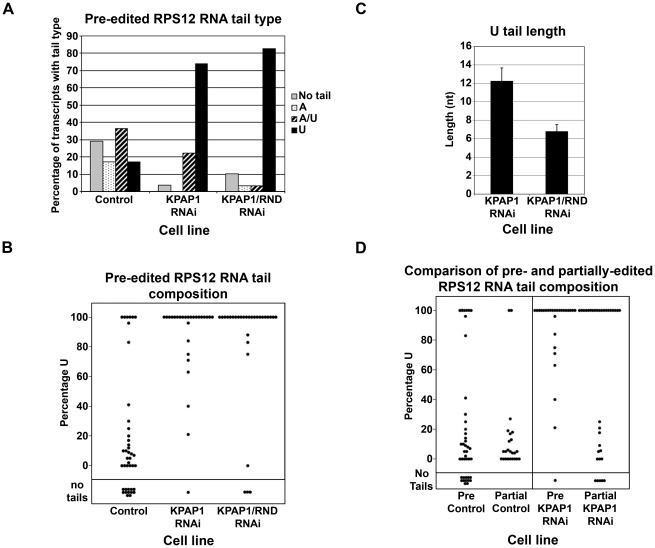
Analysis of pre- and partially-edited RPS12 RNA non-encoded tails. A. Percentage of the total population that possesses each tail type for RPS12 pre-edited transcripts in cells collected 3 days post-induction of RNAi and in uninduced cells. Total number of tails in each of the three populations ranged from 29 to 41. B. For each non-encoded tail obtained from each cell type for the pre-edited RPS12 transcripts, the percentage U in the tail was calculated and plotted. “Control” population is comprised of sequences derived from RNA of *uninduced* cultures of the TbRND, KPAP1, and KPAP1/TbRND RNAi cell lines. C. Average length of U tail in indicated cell lines with standard error shown. D. Same as B, except comparing the percentage U in tails from RPS12 transcripts prior to editing to those that were in the process being edited from both Control and KPAP1 RNAi cells. Pre-edited KPAP1 RNAi data was transposed from B for comparison. Tails in the control population for partially-edited RPS12 were obtained only from KPAP1 RNAi *uninduced* cells only.

In contrast to the never-edited ND4 RNA, RPS12 RNA undergoes extensive editing along almost the entire length of the RNA. The pre-edited version of RPS12 RNA was 1.8-fold stabilized by KPAP1 depletion, and thus differs from ND4 RNA in this regard. However, pre-edited RPS12 RNA is similar to ND4 RNA in that it is significantly stabilized (>2-fold) by dual KPAP1/TbRND depletion compared to KPAP1 depletion alone (Fig. 2A). To determine if 3′ tail composition could account for this effect, we examined pre-edited RPS12 RNA 3′ tail sequences by cRT-PCR. Since editing proceeds in a 3′ to 5′ direction, we positioned a cPCR primer just upstream of the first edited site (Fig. S1); thus, the sequences recovered would not only reveal the tail composition, but the lack of editing at site 1 would verify that the tail originated from a transcript that had not experienced editing. In control cells, the composition of 3′ tails on pre-edited RPS12 RNA is very similar to those of ND4 RNA (compare Figs. 3A and 4A), comprising relatively comparable levels of non-tailed, A tailed, U tailed and A/U tailed RNAs, with the latter predominating. Upon KPAP1 RNAi, the amount of A and A-rich tails as a percentage of the total was reduced as expected and similar to what was observed with ND4 RNA. Concurrently, the percentage of RNAs with oligo(U) tails on pre-edited RPS12 RNA skyrockets from 17% to 74% of the total population, and non-tailed RNAs are nearly eliminated (Figs. 4A and B). The effect of KPAP1 depletion on the distribution of 3′ tail sequences differs markedly between pre-edited RPS12 and the never-edited ND4 RNAs, demonstrating that pre-edited RPS12 RNAs are significantly more susceptible to 3′ uridylation than are ND4 RNAs (compare Figs. 3B and 4B). The loss of A-rich tails and the accumulation of oligo(U) tails is correlated with a modest accumulation of pre-edited RPS12 RNA (Fig. 2A). From these data, we cannot distinguish between a destabilizing effect of A-rich tails on a subset of RPS12 pre-edited RNAs or a stabilizing effect of oligo(U) tails, although the former is consistent with *in vitro* decay assays [23].

Upon co-depletion of KPAP1 and TbRND, the distribution of RNAs with different classes of tails remains relatively unchanged compared to the single KPAP1 knockdown. However, we noticed that the oligo(U) tails in the double knockdown line appeared generally shorter than those in the KPAP1 single knockdown, prompting us to sequence a larger number of clones to generate statistically significant data. These analyses revealed that 3′ tails on RPS12 pre-edited RNA in the double knockdown cell line averaged 6.7 nt in length compared to 12.2 nt in length for the tails from KPAP1 RNAi cells, and this difference was statistically significant (p<0.01) (Fig. 4C), while oligo(U) tails in the control were not abundant enough to analyze. Thus, the absence of a significant increase in the percentage of uridylated RNAs along with the decrease in oligo(U) tail length upon TbRND depletion in the KPAP1 background again suggests that TbRND-mediated decay is not directly responsible for the increased stability of pre-edited RPS12 in the dual knockdown line.

### Effect of RPS12 3′ tails on RNA editing

To this point, we have shown that pre-edited RPS12 RNA is stabilized upon KPAP1 depletion (Fig. 2A) and that this RNA population is highly enriched for oligouridylated RNAs and depleted of adenylated RNAs (Fig. 4A). Thus, we asked whether the increase in pre-edited RPS12 RNA in the KPAP1 depleted cell line was due to increased stability or decreased entry into the editing process. That is, does the composition of the 3′ tail affect the ability of an RNA to become edited? To approach this question, we asked whether RPS12 transcripts that had begun editing had the same high percentage of oligo(U) tails upon depletion of KPAP1 as the pre-edited population. A comparable ratio of tail populations in pre-edited and partially edited RPS12 would indicate that its 3′ non-encoded tail does not impact the ability of RPS12 RNA to undergo editing. To obtain partially edited RPS12 RNAs for this analysis, we utilized primers annealing to the 5′ and 3′ ends of the RNA corresponded to unedited and edited sequence, respectively (Fig. S1). We then compared the tail composition of partially edited RNAs in control and KPAP1 depleted cells to that of the previously described pre-edited RNA populations in these cell lines (Fig. 4D). In control cells (Fig. 4D, left), we observed that non-tailed RNAs constitute a large fraction of the pre-edited RNA population, while they are absent from the partially edited RNA pool. These data suggest that either adenylated RNAs are preferentially recruited to the editing pathway or that non-tailed RNAs are rapidly acquire A or AU tails once they enter the editing pathway. In KPAP1 depleted cells, pre-edited RPS12 RNAs are overwhelmingly uridylated (Figs. 4B and D). Interestingly, we found that partially edited RNAs in this cell line were also primarily uridylated, indicating that oligo(U) tails are not inhibitory to entering the editing process. However, comparison of pre-edited and partially edited RPS12 RNAs in the KPAP1 RNAi background revealed that the partially edited population was enriched for RNAs bearing A and A-rich tails compared to the pre-edited population. These data are consistent with those from the control cells, suggesting that RNAs with A-rich tails are preferentially recruited to the editing machinery. The accumulation of pre-edited RPS12 with U-rich tails in KPAP1 depleted cells might be due in part to their inefficient entry into the editing pathway.

### Effects of KPAP1, TbRND, and KPAP1/TbRND depletion on edited MURF2 RNA 3′ tail composition

**Figure 5:**
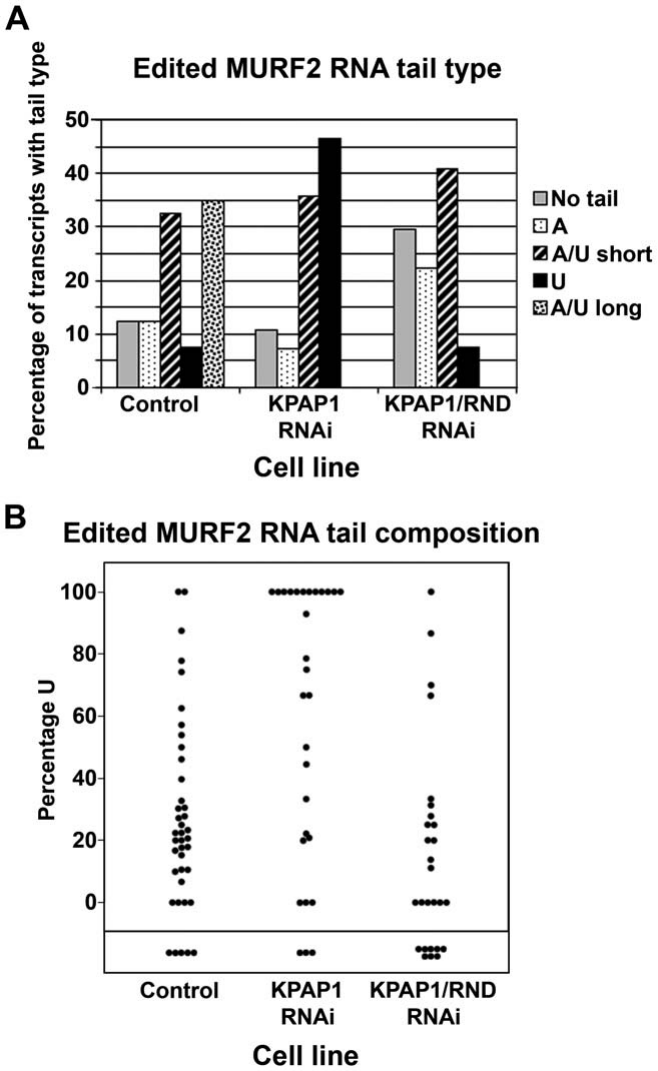
Analysis of edited MURF2 RNA non-encoded tails. A. Percentage of the total population possessing each tail type in cells collected 3 days post-induction of RNAi and in uninduced cells. Total number of tails in each of the three populations ranged from 17 to 26. B. For each non-encoded tail obtained from each cell type, the percentage U in the tail was calculated and plotted. “Control population” is comprised of sequences derived from RNA of *uninduced* cultures of the TbRND, KPAP1, and KPAP1/TbRND RNAi cell lines.

Lastly, we examined the 3′ tail composition of a transcript for which TbRND and KPAP1 co-depletion resulted a decrease rather than an increase in abundance. Edited MURF2 RNA abundance as a percentage of the total was essentially unchanged by TbRND RNAi, decreased 30% by KPAP1 RNAi, and decreased 65% by TbRND/KPAP1 co-depletion (Fig. 2). To correlate edited MURF2 RNA 3′ tails with the abundance of this RNA in the different cells lines, we subjected MURF2 RNAs to cRT-PCR using primers just 3′ of the final two editing sites. However, upon sequencing the resulting cDNAs, we found that only 65% resulted from fully edited RNA. The remaining 35% consisted primarily of RNAs with junction sequence; *i.e.*, U addition and deletion patterns that did not match the fully edited sequence [10]. We sequenced numerous fully and partially edited and noted that results from the different populations were almost indistinguishable. Thus, we combined the data for all MURF2 RNAs in Fig. 5. In this context, “edited” refers to a combined fully and partially edited RNA pool. Regarding the characteristics of 3′ tails on edited MURF2 RNAs in control cells, we found the population reminiscent of that observed in the other RNAs examined here, comprising a mix of non-tailed, A tailed, U tailed and A/U tailed RNAs. Notable differences are an increased proportion of short A/U tails and the presence of “long A/U tails”, similar to those described on MURF2 RNAs by Aphasizheva, *et al.* [18]. For this study, we defined a long tail as one with a largely homopolymeric A 5′ region, followed by an A/U rich tail, the sum of which was 40 nt or longer. Such tails often contained a short oligo(U) stretch before the A/U stretch as observed in previous sequencing of this transcript [18]. Long tails were found primarily on fully edited RNAs, although in 4 cases we observed such a tail on a partially edited RNA (Dataset S1). Short A/U tails were defined as 39 nt or shorter, and these also often contained a U homopolymer followed by an A or A/U stretch. This 5′ U homopolymer was less frequently observed with ND4 or RPS12 RNAs, thus highlighting the transcript specific differences in 3′ tail composition.

Upon depletion of KPAP1, the percentage of U-tailed edited MURF2 RNAs increased dramatically (Fig. 5). This is very similar to what we observed with pre-edited RPS12 RNA (Fig. 4), and different from ND4 RNA (Fig. 2). In addition, long A/U tails were absent from KPAP1 depleted cells. Remarkably, in complete contrast to the other two RNAs examined here, depletion of TbRND in the KPAP1 background lead to an almost complete disappearance of U-tailed RNAs. We also observed a decrease in the percentage of U in A/U tails in the dual knockdown cells compared to KPAP1 knockdowns. The population of U-tailed edited MURF2 RNAs that proportionally increases upon KPAP1 depletion (Fig. 5) appears to be destabilized upon co-depletion of KPAP1/TbRND (Fig. 2A). The coincident disappearance of U-tailed edited MURF2 RNAs (Fig. 5) and the substantial destabilization of the total edited MURF2 RNA population (Fig. 2A) indicate that TbRND is needed to maintain the U-tailed edited MURF2 RNA population. This is unexpected because, as mentioned above, TbRND has been shown to degrade U tails and promote degradation of uridylated gRNAs [26]. Collectively, these observations lead us to conclude that the ability of TbRND to promote uridylation and stability of edited MURF2 RNA is very likely an indirect effect.

## Discussion

The present study provides insights into the relationship between 3′ non-encoded tails and mRNA turnover in the mitochondria of *T. brucei*, and examines a potential role for the exoribonuclease, TbRND, in mRNA decay. Our results reveal that the effects of 3′ tails on mitochondrial mRNA stability in this organism are not as simple as previously anticipated. Oligo(A) tails have been implicated as a stabilizing element for never-edited RNAs and for edited RNAs that have begun the editing process [21], [22]. However, we had no idea of the mechanism whereby RNAs are turned over when adenylation is downregulated. It is likely that 3′ extensions are involved, but the nature of 3′ tails upon KPAP1 depletion was not known. Whether uridylation proceeds in the absence of polyadenylation, or whether tails would simply not exist in this cell line was addressed in this study. Differences in the nature of the remaining tails might explain the differential abundance changes that result upon KPAP1 depletion. It has been previously shown for one transcript that U/A addition is regulated by an accessory factor [18], so it was entirely possible that degree of uridylation upon KPAP1 depletion could be transcript specific. Our study of the ND4 and edited MURF2 transcripts (Figs. 2, 3, and 5) supports the concept that A-rich or oligo(A) tails are stability factors for edited and never-edited RNAs, at least for these RNAs. ND4 and edited MURF2 RNAs are moderately destabilized in KPAP1 depleted cells, and the oligo(A)-tailed portions of these two RNA populations disappear, suggesting that those RNAs that were adenylated required this element for their stability. Interestingly, though, non-tailed RNAs do not appear to be inherently unstable since they comprise an increased proportion of total ND4 RNAs in KPAP1 and KPAP1/TbRND knockdown cells, consistent with the fact that stabilized ND1 transcripts in RET1 depleted cells also often lack tails [17]. These results suggest that additional stability determinants, and/or, spatial segregation of sub-populations of a given RNA also contribute to its stabilization. Additional support for this conclusion arises when we analyze edited MURF2 RNA by integrating total transcript abundance (found in Fig. 2) and data from percentage of each tail type (found in Fig. 5A and B) to arrive at an approximation of abundance of RNA bearing poly(A)-rich tails (defined here as tails comprised of at least 70% [36]). We find that the abundance of these tails does not change between KPAP1 depleted and KPAP1/TbRND co-depleted cells, despite a substantial decrease in the abundance of this transcript in total (Fig. 2). This suggests that the population that is destabilized includes everything except those RNAs bearing A-rich tails, and supports the conclusion that oligo(A) and A-rich tails are not the only elements that stabilize edited and never-edited RNAs. In contrast to edited and never-edited RNAs, many pre-edited RNAs accumulate upon KPAP1 depletion ([21] and this study, Fig. 2A), supporting previous results showing oligo(A) tails destabilized pre-edited RNAs *in vitro* [23]. However, our detailed studies of pre-edited RPS12 RNA (Fig. 4) suggest that both stabilization by uridylation and decreased entry into the editing pathway may also contribute to the accumulation of pre-edited RNAs in a KPAP1 depleted background. In principle, the latter may also contribute to decreases in edited RNAs upon KPAP1 knockdown, but it cannot account for destabilization of never-edited RNAs in these cells. Regarding the role of uridylation in RNA decay, a previous study demonstrated that knockdown of RET1 resulted in dramatic increases of the never-edited transcripts MURF1 and ND1, suggesting that uridylation could be destabilizing for an mRNA [17]. Since ND1 was subsequently found to have either no 3′ non-encoded tail or a few Us in RET1 knockdown cells, it was hypothesized that the lack of U residues at the ends of MURF1 and ND1 led to their stabilization in these cells [17]. However, we find that oligo(U) tailed RNAs generally appear quite stable, exceptionally so in the case of pre-edited RPS12 RNA (Fig. 4). Therefore, U tails cannot be considered universally destabilizing. Taken together, our results demonstrate that adenylation and uridylation differentially impact the stabilities of mitochondrial RNAs in a transcript-specific fashion, and suggest that additional factors also affect mitochondrial RNA stability.

Given the substantial and transcript-specific impacts of 3′ tails on mRNA stability, it stands to reason that RNAs may be differentially susceptible to 3′ end modification. We were able to demonstrate this, as KPAP1 depletion lead to a predominance of non-tailed ND4 RNAs, but caused a very dramatic increase in the proportion of oligo(U) tailed pre-edited RPS12 and edited MURF2 RNAs. Thus, the latter two RNAs appear much more susceptible to U tail addition. Furthermore, a previous study showed that pre-edited COIII RNA appears to be primarily uridylated even in the presence of KPAP1 [19]. Together, the available data reveal that U tails are present on multiple types of mRNAs, including pre-edited, edited, and never edited classes of mRNAs, suggesting that his modification has a broad role that is not confined to one of these three populations. We hypothesize that the multitude of mainly uncharacterized mitochondrial RNA binding proteins [37] may be specifying uridylation and adenylation activity that forms the short, stability-modulating 3′ tails on mRNAs of various transcripts, in much the same way that PPR1 modulates the activity of RET1 and KPAP1 to form long tails on some never-edited and fully-edited transcripts [18], [38]. These transcript-specific factors may be differentially associated both with the RNAs and with the 3′ end-modulating enzymes. Identification of at least some of these factors could be extremely helpful in sorting out the mechanisms of transcript-specific preference of A or U addition. It is also interesting to note that ND4 is a never-edited transcript, while the uridylation-susceptible RPS12 and MURF2 RNAs both undergo editing. Thus, preferential uridylation of edited RNAs could also result from association with the editing machinery, perhaps through a transient editosome-RET1 interaction [39].

**Figure 6:**
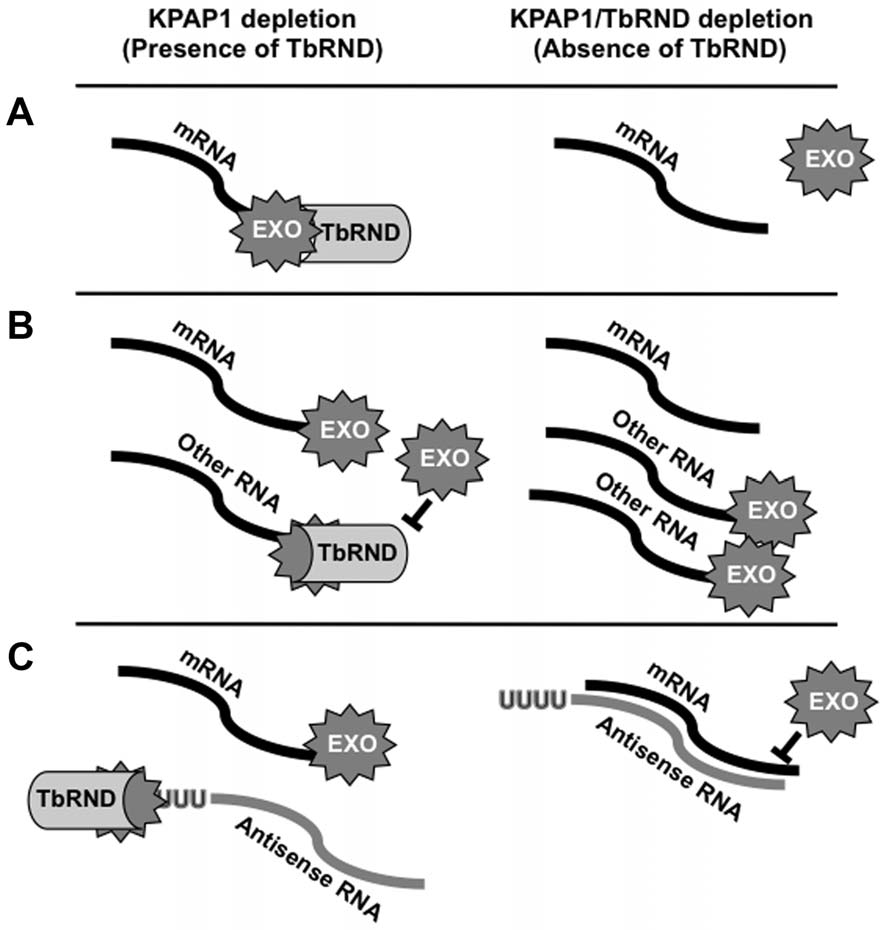
Three models explaining indirect stabilizing effects of TbRND depletion on mRNAs in a KPAP1-depleted background. A. TbRND recruits exoribonucleases to nonadenylated transcripts. B. Upon TbRND/KPAP1 co-depletion, RNAs that are normally targets of TbRND build up and generally dilute the effects of mitochondrial ribonucleases on other transcripts, thus stabilizing them. C. TbRND degrades uridylated antisense transcripts that stabilize an mRNA.

The exoribonucleases that participate in mRNA decay in trypanosome mitochondria remain elusive. Another question addressed in this study is whether TbRND participates directly in decay of some of these transcripts. Because TbRND is specific for oligo(U) [26], it has the potential to initiate degradation of oligo(U) tailed RNAs. We reasoned that oligouridylated RNAs might be more abundant in KPAP1-depleted cells, and we demonstrate that this is the case for three RNAs. If the above scenario were correct, then we would expect the oligouridylated RNA populations to be stabilized in KPAP1/TbRND co-depleted cells compared to KPAP1 single knockouts. Indeed, most transcripts are more abundant in the co-depleted cells (Fig. 2). However, the results of our 3′ tail analysis of ND4 and pre-edited RPS12 RNAs do not support a direct role for TbRND decay of these transcripts, since in neither case do oligo(U) tailed RNAs build up upon TbRND co-depletion. Moreover, the oligo(U) tails we do observe on pre-edited RPS12 are demonstrably shorter upon TbRND co-depletion, contrary to what we observe with gRNA oligo(U) tails when TbRND is knocked down [26]. Therefore, we suspect that the effects of TbRND depletion on RNA abundance and tail length are indirect. We postulate several mechanisms whereby TbRND depletion could result in the observed stabilization of many transcripts, all involving the action of yet-unidentified ribonucleases. First, TbRND could bind RNAs and recruit other nucleases to degrade them (Fig. 6A). We have performed filter binding assays and determined that TbRND displays an apparent K_d_ for mRNAs similar to that for oligo(U)-tailed gRNAs that are known to be substrates (results not shown). However, TbRND binding to mRNA does not appear to be influenced by the presence or composition of a 3′ tail, so in this scenario, the specificity of recruitment to a particular RNA would be due to factors other than the 3′ tail. Alternately, TbRND in the KPAP1 RNAi line could be directly degrading an RNA we have not selected for further study here. Upon TbRND co-depletion, this transcript would accumulate and might then sequester the majority of the ribonuclease that was previously degrading transcripts such as ND4 and pre-edited RPS12 (Fig. 6B). Indeed, while many transcripts are stabilized upon co-depletion of these two enzymes, others such as edited MURF2 are profoundly destabilized. However, if this is the explanation for these observations, both the *cis*- and *trans*-acting factors responsible for the change in substrate preference of these ribonucleases are completely unknown. Finally, TbRND depletion could result in an overabundance of an RNA population, normally a TbRND target, that directly stabilizes transcripts such as ND4 and pre-edited RPS12 (Fig. 6C). Indeed, the existence of a population of uridylated, noncoding RNAs involved in stability and processing has been previously hypothesized [17]. However, we would expect that TbRND-induced depletion of such transcripts would impact mRNA stability independent of KPAP1 level, yet TbRND's effects on mRNA stability are only apparent upon KPAP1 RNAi. Additional studies will be needed to identify the mechanisms by which TbRND can impact mRNA abundance.

Our ability to modulate the composition of mRNA 3′ tails also allowed us to address whether such extensions on pre-edited RNAs can affect the ability of these RNAs to enter the editing pathway (Fig. 4D). This is important because our results from control populations indicate that a given RNA can have multiple different tail types, so altering the ratios of different tails could provide a mechanism for regulation of RNA editing. We find that oligo(U)-tailed RNAs can efficiently undergo editing. However, it is striking that partially-edited RPS12 RNAs are enriched in oligo(A) or A-rich tails compared to the population of the fully pre-edited population, under both normal conditions and KPAP1 depletion (Fig. 4D). A/U tails with a high U content appear to be strongly selected against for entry into the editing pathway. There are two possible explanations for this observation. First, transcripts with oligo(A) or A-rich tails may be preferentially selected by the editing machinery or accessory factors to undergo editing. If this is the case, the depletion of these tail types in KPAP1 depleted cells may contribute to the decreased abundance of edited transcripts in these cells [21]. An alternate explanation for the increased percentage of A residues in 3′ tails from partially-edited RPS12 is that KPAP1, being associated with editing accessory factors [40], tends to be more concentrated around RNA undergoing editing than around RNA that is not yet associated with the editing machinery or associated complexes. But if the latter scenario is correct, we would expect that many pre-edited RNAs would bear tails with 5′ U-rich sequences followed by A-rich stretches added by editing-associated KPAP1, and this is not observed (Dataset S1). Future experiments will reveal whether preferential association of oligo(A) and A-rich tails with active editing is widespread or confined to the RPS12 transcript. Determining the impact of 3′ tails on RNA editing and the potential for KPAP1 (and RET1) action on 3′ ends while editing is ongoing is a subject begging for more extensive study.

This study reveals the complicated interplay between mRNA 3′ end modifying enzymes, their target RNAs, and the ribonucleases that mediate RNA decay. Although TbRND does not appear to degrade mRNAs or their tails, the dual-depletion system utilized here remains a way to modulate both mRNA stability and tails to examine the relationship between the two. However, our complex results compellingly demonstrate the limits of our understanding imposed by the fact that we have not identified the major players in the mRNA decay pathway: the ribonucleases. While the depletion of enzymes that end-modify RNA can be a component of RNA stability research, a crucial next step will be the identification of these ribonucleases.

## Supporting Information

Figure S1 **Circular RT-PCR primer locations.** Relevant portions of sequences from ND4, RPS12, and MURF2 mRNAs are shown with primer locations indicated by arrows. Red arrows indicate reverse transcription primers, blue arrows indicate PCR primers, and the turquoise arrow indicates a nested PCR primer.
(PDF)

Dataset S1 Spreadsheet containing 3′ non-encoded tails from the various cell lines used in this study. Tails acquired from the four transcripts examined are categorized in separate sheets of the Excel file, and categorized by different cell lines within each sheet. The dataset for the MURF2 transcript also includes a designation of whether or not the tail was acquired from a completely edited MURF2 transcript.
(XLS)
