# Supplementary material for: Additive and Transcript-Specific Effects of KPAP1 and TbRND Activities on 3′ Non-Encoded Tail Characteristics and mRNA Stability in Trypanosoma brucei
Source: PLoS One. 2012 May 21;7(5):e37639. doi: 10.1371/journal.pone.0037639 (PMC3357391; doi:10.1371/journal.pone.0037639)
Supplement: Figure S1 — Circular RT-PCR primer locations. Relevant portions of sequences from ND4, RPS12, and MURF2 mRNAs are shown with primer locations indicated by arrows. Red arrows indicate reverse transcription primers, blue arrows indicate PCR primers, and the turquoise arrow indicates a nested PCR primer. (PDF) [file pone.0037639.s001.pdf]

Figure S1: Circular RT-PCR Primer Locations

ND4

```
1     ATGTTAAAAT TAAATTTAAT ATGTATAAAT TTTATATTGT TAATTGTTAC

51    AATAATATAT ATATATATAA ACTATAGTTT TTGTATTGGA ATAGAAATCA

101   ATTATGTATA TGTAAATATA TATTTAAATT ACATCAGTCT ATGATTTGTA

151   TTTTTTATGG GAATTATTAT .................................1080 nt...........................

1251  TGTATTTATG TCAATATCAA TATCAACTAT AGTATTTTAT TATTTTATAT

1301  ATTTATTAAT ATAA
```

RPS12 Top row: pre-edited DNA; arrows on top represent primers to pre-edited sequence
Bottom row: fully-edited RNA; arrows below represent primers to edited sequence

```
  1 ---------+---------+---------+-- - -              +          44
    CTAATACACTTTTGATAACAAACTAAAGTAAA A A     G         GCG A G GATT
    CUAAUACACUUUUGAUAACAAACUAAAGUAAAuAuAuuuuGuuuuuuuuGCGuAuGuGA U

 45 ----- + ---   -   -    ----     +    -    -     - -   -   - - --+ ------   76
    TTTTG A GTG   G   G    ACTG     G    A    G     A G   A   A A GAG CCGTTC
    UUUUGuAuG GuuGuuGuuuAC GuuuuGuuuuAuuuGuuuuAuGuuAuuAuAuGAGuCCG  C
 61 ---------+---------+---------+---------+---------+---------  +  120

 77 --  --+----  ---- --------- - -  - + ---------+ -     -  -- - --    -  118
    GA  GCCCAG   CCGG AACCGACG G A  G A GCTTCTTTTG A     A  TA A AA    G
    GAuuGCCCAGuuCCGGuAACCGACGuGuAuuGuAuGC   C    GuAuuuuAuuUAuAuAAuuuuG
121 ---------+---------+---------+-------   -    -+---------+---------+  180

119    -+- -  ---  -         -  -    -  +-   --  - -----+  -    -  -  143
       GGA G  GCG  G         G  G    A  GG   AG  A GTTTCA  A    A  A
    uuuGGAuGuuGCGuuGuuuuuuuuGuuGuuuuAuuGGuuuAGuuAuG  UCAuuAuuuAuuA
181 ---------+---------+---------+---------+-------  --+---------+  240

144  ------+----- --    -  -+   ---------+--- ------+ -  - -------+--  - ---
     AGATTTGGGTGG GG    G  GA   ACCCTTTGTTTTG GTTAAAG A  A ACATCGTTTA  G AAG
    uAGA   GGGUGGuGGuuuuGuuGAuuuACCC    G    GuG UAAAGuAuuAuACA CG  UAuuGuAAGuu
241 ----   -----+---------+---------    +    --- ------+------- --  +---------+

197 -+-------- - ---+---------+-  221
    AGATTTTAGA ATAAGATATGTTTTT
    AGA UUUAGAuAUAAGAUAUGUUUUU
301 --- ------+---------+-----  325
```

PCR Primer Location

RT Primer Location

Supplemental Figure 1: Circular RT-PCR Primer Locations

MURF2

Top row: pre-edited DNA
Bottom row: fully-edited RNA; arrows below represent primers to edited sequence or sequence of a region of the gene that does not undergo editing
Start codon is boxed

```
   1 ---------+---------+---------+--    -- -   --  -    -+----   --     46
     TTTTATATAGAAAGGTATATAATCTATAATGA    AA G   GG  G    ATTTTA   AG
     UUUUAUAUAGAAAGGUAUAUAAUCUAUAAUGAuuuuAAuGuuuGGuuGuuuuA    AuuuAGu
   1 ---------+---------+---------+---------+---------+---------+---   60

  47     -   --+ ---------+ ---------+---------+---------+--------  98
         A   TTG GCTTTGATTG AGTCGTGTTTTTGATTTGTTATGTATTAGAACATATGA
      uuuAuuuUUGuGCUUUGAUUGuAGUCGUGUUUUUGAUUUGUUAUGUAUUAGAACAUAUGA
  61  --------+---------+---------+---------+---------+---------+  120

  99 -+---------+---------+-----
     TTTTATATTGTGATGGTTTGATCTAGA
     UUUUAUAUUGUGAUGGUUUGAUCUAGA
 121 ---------+---------+-------.............873 nt..........................

 999 -+---------+---------+---------+---------+---------+-------- 1058
     ATTATATAATTACGATATATTATCATATAGTATATTTTATTATCAAAATAATCAGTTTTG
     AUUAUAUAAUUACGAUAUAUUAUCAUAUAGUAUAUUUUAUUAUCAAAAUAAUCAGUUUUG
1021 ---------+---------+---------+---------+---------+---------+ 1080

1059 -+---------+---------+------ 1086
     TTTAACACAGTTATTATCAATTTATATA
     UUUAACACAGUUAUUAUCAAUUUAUAUA
1081 ---------+---------+-------- 1108
```

Nested PCR Primer Location

PCR Primer Location

RT Primer Location
